# Expanded characterization of *in vitro* polarized M0, M1, and M2 human monocyte-derived macrophages: Bioenergetic and secreted mediator profiles

**DOI:** 10.1371/journal.pone.0279037

**Published:** 2023-03-02

**Authors:** Elise Hickman, Timothy Smyth, Catalina Cobos-Uribe, Robert Immormino, Meghan E. Rebuli, Timothy Moran, Neil E. Alexis, Ilona Jaspers

**Affiliations:** 1 Center for Environmental Medicine, Asthma, and Lung Biology, The University of North Carolina at Chapel Hill, Chapel Hill, North Carolina, United States of America; 2 Curriculum in Toxicology & Environmental Medicine, The University of North Carolina at Chapel Hill, Chapel Hill, North Carolina, United States of America; 3 Department of Pediatrics, The University of North Carolina at Chapel Hill, Chapel Hill, North Carolina, United States of America; University of Alabama at Birmingham, UNITED STATES

## Abstract

Respiratory macrophage subpopulations exhibit unique phenotypes depending on their location within the respiratory tract, posing a challenge to *in vitro* macrophage model systems. Soluble mediator secretion, surface marker expression, gene signatures, and phagocytosis are among the characteristics that are typically independently measured to phenotype these cells. Bioenergetics is emerging as a key central regulator of macrophage function and phenotype but is often not included in the characterization of human monocyte-derived macrophage (hMDM) models. The objective of this study was to expand the phenotype characterization of naïve hMDMs, and their M1 and M2 subsets by measuring cellular bioenergetic outcomes and including an expanded cytokine profile. Known markers of M0, M1 and M2 phenotypes were also measured and integrated into the phenotype characterization. Peripheral blood monocytes from healthy volunteers were differentiated into hMDM and polarized with either IFN-γ + LPS (M1) or IL-4 (M2). As expected, our M0, M1, and M2 hMDMs exhibited cell surface marker, phagocytosis, and gene expression profiles indicative of their different phenotypes. M2 hMDMs however were uniquely characterized and different from M1 hMDMs by being preferentially dependent on oxidativte phosphorylation for their ATP generation and by secreting a distinct cluster of soluble mediators (MCP4, MDC, and TARC). In contrast, M1 hMDMs secreted prototypic pro-inflammatory cytokines (MCP1, eotaxin, eotaxin-3, IL12p70, IL-1α, IL15, TNF-β, IL-6, TNF-α, IL12p40, IL-13, and IL-2), but demonstrated a relatively constitutively heightened bioenergetic state, and relied on glycolysis for ATP generation. These data are similar to the bioenergetic profiles we previously observed in vivo in sputum (M1) and BAL (M2)-derived macrophages in healthy volunteers, supporting the notion that polarized hMDMs can provide an acceptable *in vitro* model to study specific human respiratory macrophage subtypes.

## Introduction

Macrophages play vital roles in maintaining immune homeostasis in the large and distal airways. Key macrophage functions include phagocytosis of microbial pathogens, release of cell signaling molecules, and participation in tissue remodeling [1–3]. It is well-established that macrophages exhibit functional and cell surface phenotype plasticity that can be altered depending on their location within the respiratory tract and in response to stimuli present in their microenvironment [3–5]. There is also emerging appreciation for the important role that cellular metabolism (bioenergetics) plays in mediating macrophage activation and polarization that drive both subsequent innate and acquired immune responses [6, 7]. For example, when macrophages encounter pro-inflammatory or pathogenic stimuli, they undergo a metabolic shift from mitochondrial-based oxidative phosphorylation pathways to glycolysis-based pathways, thereby allowing the cell to rapidly respond to immediate increased energy demands during active infection or inflammation [8–10].

Many factors limit the routine experimental use of airway macrophages collected from human volunteers. Whether it is by induced sputum for central airways cells, or from bronchoalveolar lavage (BAL) for distal airway cells, both sampling techniques require specific expertise, are time consuming, involve a degree of subject risk, and, for BAL in particular, is costly [11–13]. Therefore, *in vitro* models are necessary for macrophage experimentation and have been used extensively as surrogates to assess *in vivo* macrophage function and phenotype. One such model uses human monocyte-derived macrophages (hMDMs), which are isolated from the peripheral blood of human subjects and then differentiated in culture into various macrophage phenotype subsets. This model is accessible, biologically relevant, and relatively easy to use. Across studies however, there is considerable variability in culture conditions that include length of differentiation and mediator cocktails inducing differentiation and polarization. Most commonly, monocytes are differentiated over about one week with M-CSF or GM-CSF to produce naive hMDMs (M0) and then polarized into M1 and/or M2 macrophages using stimuli such as IFN-y and lipopolysaccharide (LPS) (M1) or IL-4 and IL-13 (M2), and others, depending on the desired phenotype [4, 14–16]. Previous studies have characterized the phenotypes of hMDMs polarized into M1 and M2 macrophages, with a significant focus on transcriptional changes and to a lesser extent, on markers of cell function such as cell surface receptor expression and phagocytosis [4, 15, 16]. However, characterization of bioenergetic profiles of naive hMDMs (M0) and hMDMs polarized to M1 and M2 are largely lacking.

Previously, our group observed that in healthy individuals, sputum macrophages from the surfaces of the central airways reflect an M1 glycolysis-dependent phenotype, whereas BAL macrophages recovered from the distal airways reflect an M2 oxidative phosphorylation-dependent phenotype [17]. Animal studies using MDMs have also demonstrated bioenergetic differences between subsets of polarized macrophages [18–20]. Thus, in order to determine how close *in vitro* derived hMDMs and their polarized subsets reflect in vivo macrophage phenotypes in the respiratory tract, better integration of bioenergetic profiles, cytokine release, and markers of cell function are critically needed. The overall aim of this study was to generate hMDMs in culture, polarize them into M1- and M2-like subsets, and measure cellular bioenergetics, cytokine release, cell surface marker expression, and gene expression profiles to comprehensively profile these important cells. Our data presented here indicate that M2 hMDMs were characterized by secreting a unique cluster of soluble mediators (MCP4, MDC, and TARC) and dependence on oxidative phosphorylation for their ATP generation. In contrast, M1 hMDMs secreted significantly more of prototypic pro-inflammatory cytokines (MCP1, eotaxin, eotaxin-3, IL12p70, IL-1α, IL15, TNF-β, IL-6, TNF-α, IL12p40, IL-13, and IL-2), existed in an activated state, and relied on glycolysis for bioenergetics.

## Materials and methods

### Subjects

Healthy non-smoking adult human subjects participated in a venous blood draw. Study exclusion criteria included current nicotine use, acute illness, allergy symptoms, asthma, and/or pregnant and nursing women. The sex of subjects in each experiment is reported in the figure legend. Written informed consent was obtained from all subjects, and all studies were approved by the University of North Carolina at Chapel Hill School of Medicine Institutional Review Board (IRB #11–1363).

### Monocyte isolation

Venous blood was collected in BD Vacutainer tubes with EDTA. Peripheral blood mononuclear cells were isolated using Ficoll-Paque Plus (Cytivia) density centrifugation and washed 3 times with DPBS. CD14+ monocytes were isolated using magnetic bead negative selection per the manufacturer’s protocol (EasySep Human Monocyte Isolation Kit, Stemcell Technologies). After negative selection, the purity of the resulting cell population was verified using flow cytometry, with an average of ~90% CD14+ monocytes (S1 Fig).

### Monocyte differentiation

Immediately following isolation, CD14+ monocytes were seeded at a density of 187,500 cells/cm^2^ in various sizes of tissue-culture treated multi-well plates. Monocyte base media was RPMI-1640 media (Gibco) with 10% FBS (Millipore Sigma) and 1% penicillin/streptomycin (100 U/mL, Gibco). L-glutamine (Gibco) was added to the base media immediately before cell seeding and/or feeding (2 mM final concentration). Monocytes were differentiated into naïve (M0) macrophages with base media + 40 ng/mL M-CSF. Four days after the isolation, the media was replaced. Six days after the isolation, differentiation media was removed, and cells were polarized into M1 hMDMs with 20 ng/mL IFN-y + 20 ng/mL LPS, M2 hMDMs with 20 ng/mL IL-4, or M0 hMDMs with no stimulants added to the base media. Samples were collected and phenotype assays were performed approximately 24 hours after polarization. M0, M1, and M2 hMDMs were derived from each donor such that within each experiment, matched analyses could be performed between the polarization states.

### Cytotoxicity

Cytotoxicity was determined using the CellTox Green Cytotoxicity Assay (Promega). 24 hours after polarization, hMDMs were washed once with DPBS (Gibco) and media was replaced with fresh base media. Lysis buffer was added to untreated hMDMs, and cells were incubated for 15 minutes at 37C to induce maximum cytotoxicity. Equal volumes of 2x CellTox Green dye in assay buffer were added to each well and incubated for 15 minutes at 37°C. Fluorescence was quantified using a CLARIOstar plate reader (BMG Labtech) and data were normalized by subtracting mean fluorescence intensity of 1x CellTox Green dye in base media from fluorescence intensity of each well. Cytotoxicity was presented as the percent of average lysis well FI.

### Gene expression

24 hours after polarization, hMDMs were washed with DBPS and lysed in Ambion lysis buffer with 1% β-mercaptoethanol. Lysate was stored at -80°C until samples were collected from all subjects. Total RNA was isolated using the Ambion Pure Link RNA Mini Kit (Life Technologies). RNA was reverse transcribed into cDNA as described previously [21]. Real-time quantitative PCR was performed with cDNA using Applied Biosystems TaqMan Universal Master Mix II with UNG (Thermo Fisher Scientific), TaqMan assays, and the QuantStudio3 Real-Time PCR System (Thermo Fisher Scientific). Genes were selected based on previous studies of polarized macrophages, with an emphasis on human macrophages when possible (Table 1). TaqMan assays were as follows: Hs00968979_m1 (*ARG1*), Hs00267207_m1 (*MRC1*), Hs01075529_m1 (*NOS2*), Hs00153133_m1 (*PTGS2*). Gene expression differences were calculated using the 2^-DDCt^ method [22] with *ACTB* as the endogenous control and M0-like hMDMs as the reference phenotype.

**Table 1:** Genes selected for RT-qPCR and rationale for their selection.

Gene Abbreviation	Name	Function of Encoded Protein	Rationale for Selection
*ARG1*	Arginase 1	Enzyme that hydrolyzes arginine to urea and ornithine,	Increased *ARG1* expression has been associated with M2 phenotype in murine macrophages [9, 23, 24]; however, whether this is consistent in human macrophages is unclear [16, 25].
*MRC1*	Mannose receptor C-type 1	Cell surface receptor that senses extracellular mannoglycoproteins and other pathogen-associated ligands	Increased expression in M2 murine and human macrophages [26, 27].
*NOS2*	Nitric oxide synthase 2, inducible	Enzyme that synthesizes nitric oxide	Increased expression in M1 macrophages, with a majority of studies in murine macrophages [27, 28].
*PTGS2*	Prostoglandin-endoperoxide synthase 2 (cyclooxygenase 2)	Enzyme that catalyzes the conversion of arachidonic acid into prostaglandins	Increased expression of *PTGS2* in M1 human macrophages [14].

### Cytokine secretion

24 hours after polarization, media was collected and centrifuged at 1000 x g for 10 minutes to remove any cellular debris. Supernatant was transferred to a new tube and stored at -80°C until all samples were collected. Protein concentrations were measured using commercially available single- and multi-plex ELISAs (IL-6 and IL-8: BD Bioscience; TNF-a, CCL17, CCL18, MMP-2, MMP-9: R&D Systems; V-PLEX Human Cytokine 30-plex: Mesoscale Discovery). For single-plex ELISAs, absorbance was quantified using a CLARIOstar plate reader (BMG Labtech) per assay instructions. The V-PLEX Human Cytokine 30-plex was read on the MESO QuickPlex SQ 120 (Mesoscale Discovery).

### Phagocytosis

24 hours after polarization, hMDM phagocytosis of *S*. *aureus* and Zymosan A pHrodo Red Bioparticles (Thermo Fisher Scientific) was measured as described previously [29], with a Bioparticle incubation time of 2 hours. Fluorescence in each well was quantified using a CLARIOstar plate reader (BMG Labtech).

### Cellular bioenergetics

24 hours following polarization, hMDMs were assayed for bioenergetic parameters using the Seahorse Extracellular Flux Modified Cell Mito Stress Test (Agilent) as described previously [17]. Briefly, polarization media was replaced with Seahorse XF RPMI, pH 7.4, supplemented with 2 mM L-glutamine. Then, hMDMs were incubated in a non-CO_2_ incubator for 30–40 minutes before the start of the assay. Injection order and final concentrations of treatments were as follows: Port A– 10 mM glucose; Port B– 1 mM oligomycin; Port C– 1.25 mM FCCP; Port D– 0.5 mM rotenone and 0.5 mM antimycin A. Mix-wait-measure times were 3 min– 2 min– 3 min, per manufacturer’s instructions. Mitochondrial and glycolytic parameters were calculated as described previously and as recommended by the manufacturer [17]. Immediately following the assay, nuclei were stained using Hoechst 33342 (Thermo Fisher Scientific), and fluorescence in each well was quantified using a CLARIOstar plate reader (BMG Labtech). Data were normalized by dividing bioenergetic parameters by mean Hoechst 33342 fluorescence intensity in each well.

### Mitochondrial membrane potential

Mitochondrial membrane potential was measured using JC-1 dye. JC-1 (Thermo Fisher) is a membrane permeable dye which accumulates within mitochondrial membranes in a membrane potential-dependent manner [30]. JC-1 forms red fluorescent aggregates within the membrane and mitochondrial membrane potential can be measured by determining the ration of red fluorescent aggregates and green, fluorescent monomers [30]. The mitochondrial membrane potential disruptor Carbonyl cyanide m-chlorophenyl hydrazone (CCCP, Sigma-Aldrich) was used to determine minimal red fluorescence (S2 Fig) [30]. 24 hours after polarization, hMDMs were washed with DPBS (Gibco) and 4 uM JC-1, 4 uM JC-1 with 100 uM CCCP, or base media were added to wells. Cells were incubated for 30 minutes at 37C, and media was replaced with fresh base media. Cells were incubated for an additional 10 minutes at 37C, and fluorescence was quantified using a CLARIOstar plate reader (BMG Labtech). Data were normalized by subtracting mean base media fluorescence intensity from fluorescence intensity of each well.

### Intracellular nitric oxide production

Intracellular NO production was measured using the NO reactive dye DAF-2 DA. DAF-2 DA is a membrane permeable dye which is metabolized to the membrane impermeable DAF-2 following reaction with intracellular esterases [31]. DAF-2 DA (AAT Bioquest) was reconstituted in DMSO (Sigma-Aldrich) and diluted to a final concentration of 5 uM in base media immediately prior to use. 24 hours after polarization, hMDMs were washed once with DPBS (Gibco) and DAF-2 DA in base media was added to each well. Cell-free wells were incubated with 5 uM DAF-2 DA in base media to act as a media fluorescence control. Following a 1-hour incubation at 37°C, cell and cell-free wells were washed once with DPBS and fresh DPBS was added to each well. Fluorescence was quantified using a CLARIOstar plate reader (BMG Labtech). Data were normalized by subtracting mean cell-free fluorescence intensity from fluorescence intensity of each well.

### Flow cytometry

Flow cytometry markers were chosen based on previous studies characterizing human monocyte-derived macrophages and human respiratory tract macrophages [5, 14, 15, 32, 33], with functions described in Table 2. 24 hours after polarization, hMDMs were washed three times with DPBS and dissociated via incubation (37°C, 5% CO_2_) with Cellstripper (Corning) for 30 minutes, followed by thorough washing over the well with a micropipette to aid in cell detachment. Cells were pelleted (400 x g for 5 minutes) and counted with a hemocytometer. Prior to staining, 2-5x10^5^ hMDMs were incubated with human TruStain FcX (BioLegend) for 5 min to block Fc receptors. Live/dead cell discrimination was achieved using Zombie Aqua (BioLegend). Direct fluorochrome-conjugated antibodies against CD64 (clone 10.1; PerCP-Cy5.5), CD206 (15–2; FITC), HLA-DR (L243; PE-Cy7), CD86 (IT2.2; PE), CD14 (HCD14; APC-Cy7) and CD163 (GHI/61; AF-647) were used for detection of hMDM surface markers. Following staining cells were washed and then fixed with 4% PFA in PBS. Flow cytometry data were acquired with a four-laser LSRII (BD Biosciences) and analyzed using FlowJo software. Mean fluorescence intensity (MFI) was used as a readout for surface marker expression. Only single cells were analyzed. All antibodies were purchased from BioLegend.

**Table 2:** Function of cell surface markers selected for flow cytometry.

Cell Surface Marker	Function
CD64	Also known as Fc-gamma receptor 1, binds IgG
CD86	Costimulatory signaling to promote T-cell activation and survival
CD163	Hemoglobin-haptoglobin scavenger receptor
CD206	Mannose receptor
HLA-DR	Antigen presentation

### Statistical analyses

All statistical analyses were performed in GraphPad Prism 9. For each set of data, normality was assessed using the D’Agostino & Pearson test. Normally distributed data were analyzed using matched one-way ANOVA with Tukey’s multiple comparisons test. Non-normally distributed data were analyzed using the Friedman test with Dunn’s multiple comparisons test (if no values were missing) or the Friedman test with Holm-Sidak multiple comparisons test (which allows for missing values). When possible, we investigated sex differences in hMDM function. In the cohort of samples used for single-plex ELISAs, we tested for sex differences in the expression of proteins in each treatment group using a two-way ANOVA with sex and polarization as factors. Except for MMP-9 (p = 0.0136 overall; p = 0.0639 between males and females in the M1 group, with cells from females having higher expression than cells from males), sex was not a significant source of variation in our data. We also tested for but did not detect sex differences in phagocytosis or cellular bioenergetics. Multi-plex ELISA data are displayed using a row scaled heatmap, which was generated in R version 4.1.1 [34] using the *pheatmap* [35] and *viridis* [36] packages. Raw concentrations and additional statistical comparisons for multi-plex ELISA data are available in S1 Table. To reduce dimensionality in our data and further explore differences in soluble mediator expression between subsets of polarized hMDMs, we performed Principal Component Analysis (PCA) using the R packages *ggfortify* [37, 38] and *factoextra* [39]. PCA takes a set of variables that are part of a given dataset and generates new variables (representing a combination of original variables) that attempt to contain the majority of the variation of the data in the first few new variables, also called principal components, or dimensions (Dim). Results are presented as plots of samples (unique donors and polarization states) for the first two principal components, which represent a certain amount (%) of variation in the dataset as whole (Dim % reported on each individual axis) [39]. Input data and R code used for these analyses are publicly available at https://github.com/ehickman0817/hMDM-phenotypes.

## Results

### M0, M1, and M2 hMDMs have unique bioenergetic profiles that are similar to human in vivo airway macrophages

**Fig 1:**
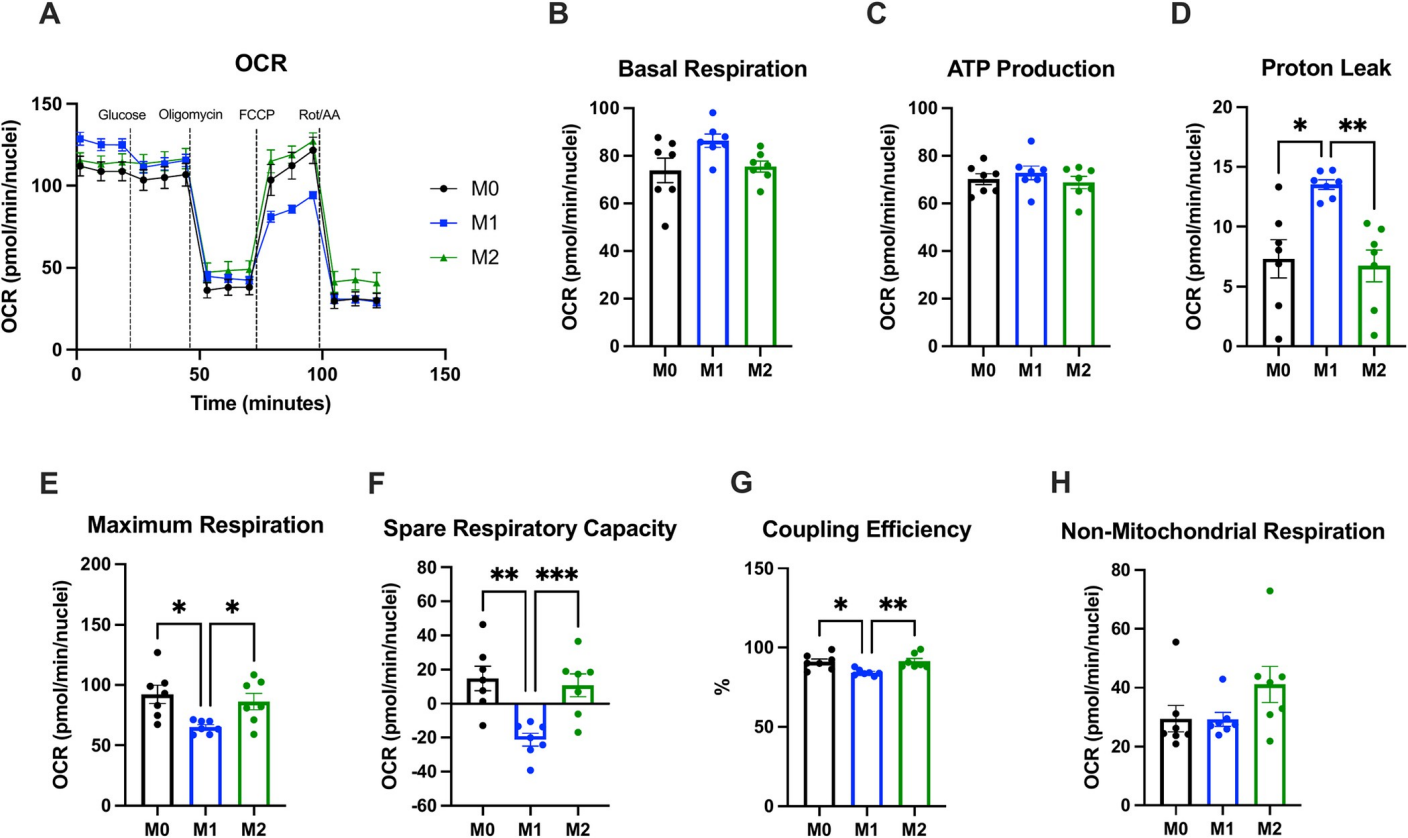
hMDM polarization induces changes in mitochondrial function. hMDM OCR (A) was measured using Seahorse Extracellular Flux. There were no significant differences between polarized hMDM subsets for basal respiration (B), ATP production (C), and non-mitochondrial respiration (H). M1 hMDMs had significantly higher proton leak (D) and significantly lower maximum respiration (E), spare respiratory capacity (F), and coupling efficiency (G) than M0 and M2 hMDMs. n = 7 biological replicates (3 males, 4 females) with 3–4 technical replicates per biological replicate and polarization state. Data are presented as mean ± SEM. * p < 0.05, ** p < 0.01, *** p < 0.001 by matched one-way ANOVA with Tukey’s multiple comparisons test.

**Fig 2:**
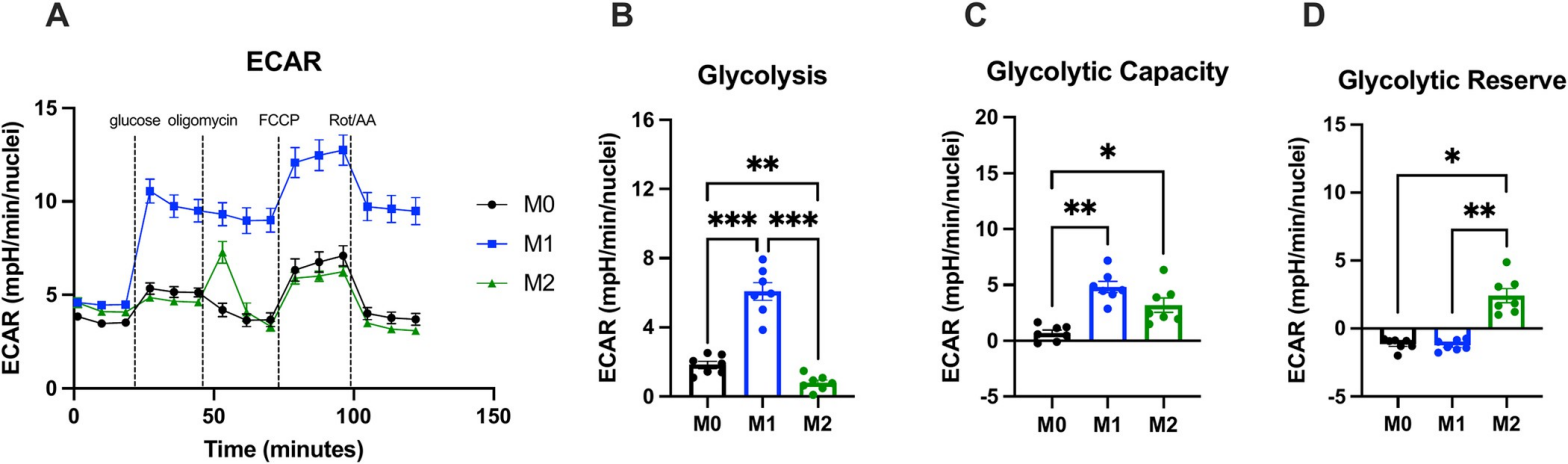
hMDM polarization induces changes in glycolysis. hMDM ECAR (A) was measured using Seahorse Extracellular Flux. M1 hMDMs were significantly more glycolytic (B) than other polarization states, and both M1 and M2 hMDMs had significantly higher glycolytic capacity (C) than M0 like hMDMs. M2 hMDMs had significantly more glycolytic reserve than M0 or M1 hMDMs (D). n = 7 biological replicates (3 males, 4 females) with 3–4 technical replicates per biological replicate and polarization state. Data are presented as mean ± SEM. * p < 0.05, ** p < 0.01, *** p < 0.001 by matched one-way ANOVA with Tukey’s multiple comparisons test (B,C) or Friedman test with Dunn’s multiple comparisons test (D).

To determine bioenergetic differences between our polarized macrophages subsets, we performed Seahorse Extracellular Flux assays, which simultaneously measure the oxygen consumption rate (OCAR) and extracellular acidification rate (ECAR) in cell media following exposure of cells to glucose and mitochondrial inhibitors (Figs 1 and 2). There were no significant differences between polarization states in basal respiration, ATP production, and non-mitochondrial respiration (Fig 1B, 1C and 1H). M1 hMDMs had significantly higher proton leak and lower mitochondrial respiration, spare respiratory capacity, and coupling efficiency than M0 and M2 hMDMs (Fig 1D–1G), indicating less efficient generation of energy/ATP via oxidative phosphorylation (OXPHOS) in the mitochondria. We also found that M1 hMDMs were significantly more glycolytic than M0 and M2 hMDMs (Fig 2B). Interestingly, both M1 and M2 hMDMs had significantly higher glycolytic capacity than M0 hMDMs (Fig 2C), and M2 hMDMs had significantly higher glycolytic reserve than M0 and M1 hMDMs (Fig 2D). Overall, these results support the notion that M1 hMDMs rely more on glycolysis and exist in a more high-energy, activated state at baseline, while M2 hMDMs rely more on oxidative phosphorylation for ATP generation and are better able to respond to increased demand for energy via OXPHOS and glycolysis.

Because we observed significantly higher proton leak in M1 hMDMs, we wanted to determine whether mitochondrial membrane potential was significantly different between polarization states. Using JC-1 dye to measure mitochondrial membrane potential, we found that there were no significant differences between polarization states (S2 Fig), suggesting that despite increased proton leak in M1 hMDMs, mitochondrial membrane potential is maintained. Next, to assess whether bioenergetic changes were caused by differences in cell viability, we assessed cytotoxicity following polarization (S3 Fig). Although M2 hMDMs had significantly lower cytotoxicity than M1 hMDMs, overall, polarization did not induce significant cytotoxicity, with mean percent cytotoxicity between 6–11% of the lysed positive control.

**Fig 3:**
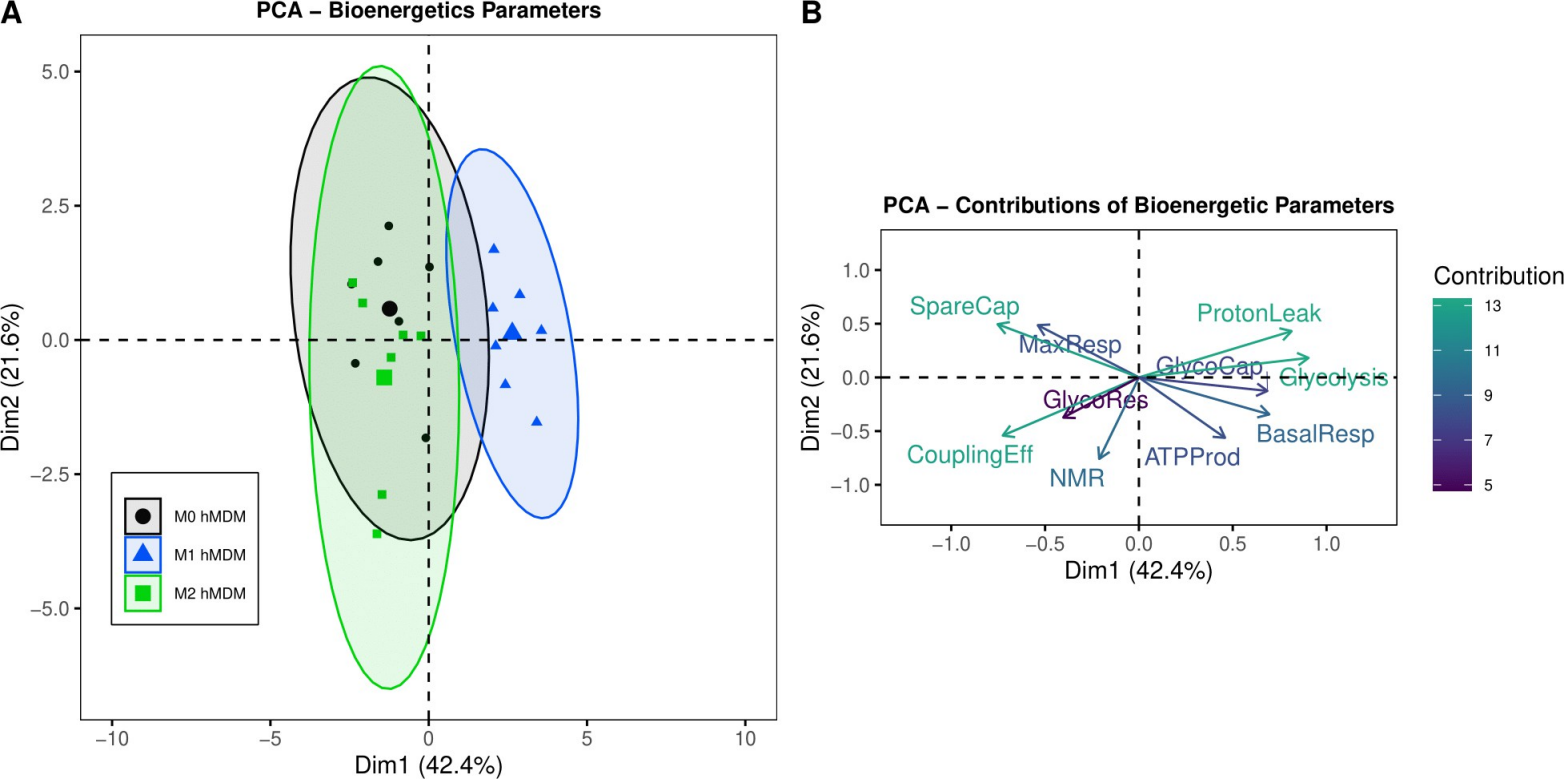
PCA analysis of Seahorse Extracellular Flux bioenergetic parameters. (A) Plot showing clustering of bioenergetic parameters by polarized hMDMs along the first two principal components (Dim1 and Dim2). (B) Percentage contributions of each variable to the variation observed in the first two PCA dimensions. n = 7 biological replicates (3 males, 4 females) with 3–4 technical replicates per biological replicate and polarization state.

To assess overall differences in bioenergetic profiles, we performed principal components analysis (PCA), including all ten bioenergetic parameters obtained with Seahorse Extracellular Flux (Fig 3). Principal component 1 (Dim1) represented 42.4% of the variation in the dataset and separated M1 hMDMs from M0 and M2 hMDMs (Fig 3A). Principal component 2 (Dim2) represented 21.6% of the variation in the dataset and did not show separation by polarization state (Fig 3A). M0 and M2 hMDMs did not cluster distinctly from each other, suggesting similar bioenergetic profiles. Plotting contributions of each variable to the separation in Fig 3A demonstrated that spare capacity, coupling efficiency, glycolysis, and proton leak contributed the most to separation of M1 versus M0 and M2 hMDMs along the first principal component axis (Fig 3B). These results suggest a unique bioenergetic profile in M1 hMDMs in comparison with M0 and M2 hMDMs, in agreement with analysis of individual bioenergetic parameters (Figs 1 and 2).

### Polarization of macrophages to M0-, M1-, and M2-like phenotypes significantly changes cytokine secretion

We measured the concentrations of secreted cytokines and immune mediators in our different macrophage subsets that have previously been shown to differentiate macrophage subtypes [14] (S1 Table). We found that M1 hMDMs secreted significantly more IL-6, IL-8, and TNF-α than M0 and M2 hMDMs (S1 Table) and that M2 hMDMs secreted significantly more CCL17 than M0 and M1 hMDMs and more CCL18 than M0 hMDMs (S1 Table), as expected. We were also interested in whether these cells secreted matrix metalloproteinases, such as MMP2 and MMP9, due to their important roles in tissue remodeling in the lungs. All hMDMs secreted MMP-9, with M1 hMDMs secreting significantly less than M0 hMDMs (S1 Table). MMP-2 was not secreted by the hMDMs.

**Fig 4:**
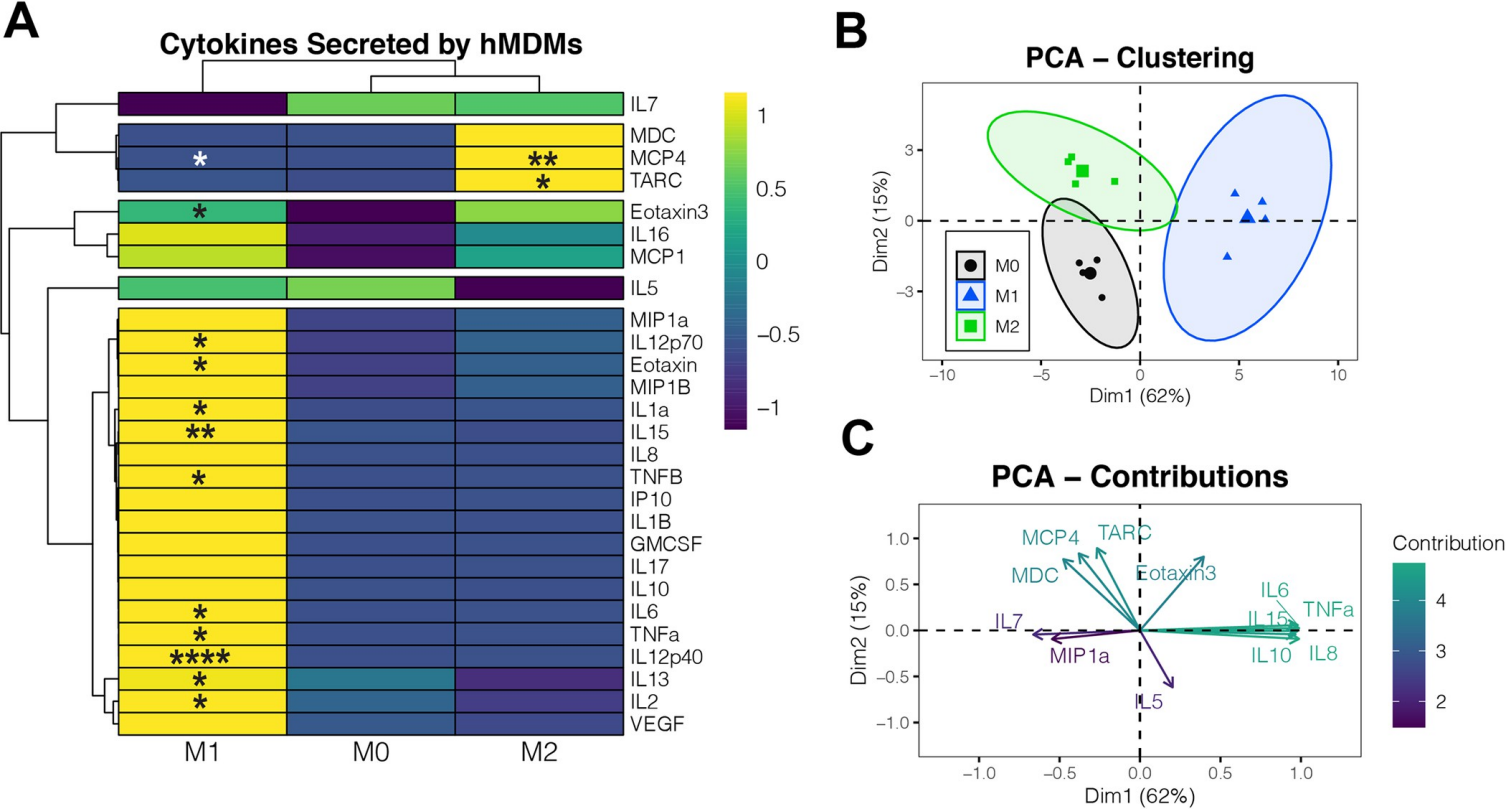
Expanded characterization of cytokines, chemokines, and soluble mediators expressed by hMDM. (A) Row-scaled heatmap showing protein expression. * p < 0.05, ** p < 0.01, **** p < 0.0001 in comparison with M0 hMDMs by either matched one-way ANOVA with Tukey’s multiple comparisons test or Friedman test with Dunn’s multiple comparisons test. Raw concentrations and significant comparisons between M1 and M2 hMDMs are reported in S2 Table. (B) PCA plot showing clustering of cytokine secretion by polarized hMDMs. (C) Percentage contributions of each variable to the variation observed in the first two PCA dimensions. For all plots, n = 4 biological replicates (1 male, 3 females) with one technical replicate per biological replicate and polarization state.

We expanded our cytokine analysis to better understand the secretome of polarized hMDMs. To that end, we measured a panel of 27 cytokines, chemokines, and other secreted mediators using multi-plex ELISA on a separate set of samples (Fig 4, S2 Table). We found that there were secreted mediators unique to each polarization state; for example, M2 hMDMs secreted high levels of MCP4, MDC, and TARC, while M1 hMDMs secreted high levels of many pro-inflammatory cytokines and chemokines (Fig 4A). In comparison with M0 hMDMs, M2 hMDMs secreted significantly more MCP4 and TARC, and M1 hMDMs secreted significantly more MCP4, eotaxin, eotaxin-3, IL12p70, IL-1α, IL15, TNF-β, IL-6, TNF-α, IL12p40, IL-13, and IL-2. PCA demonstrated clear separation between polarized hMDM subsets (Fig 4B) that are driven by differences in soluble mediators similar to those that were significantly different between groups using variable-by-variable analysis (Fig 4C). Principal component 1 (Dim1) represented 62% of the variation in the dataset and separated M0 and M2 hMDMs from M1 hMDMs, while principal component 2 (Dim2) represented 15% of the variation in the dataset and separated M0 and M2 hMDMs (Fig 4B). These data support the previously published paradigm that M1 hMDMs are more pro-secretory and provide additional data to establish baseline secretory states of polarized hMDMs [14, 40].

### Polarization of macrophages to M0-, M1-, and M2-like phenotypes significantly changes gene expression, cell surface marker expression, and phagocytosis

**Fig 5:**
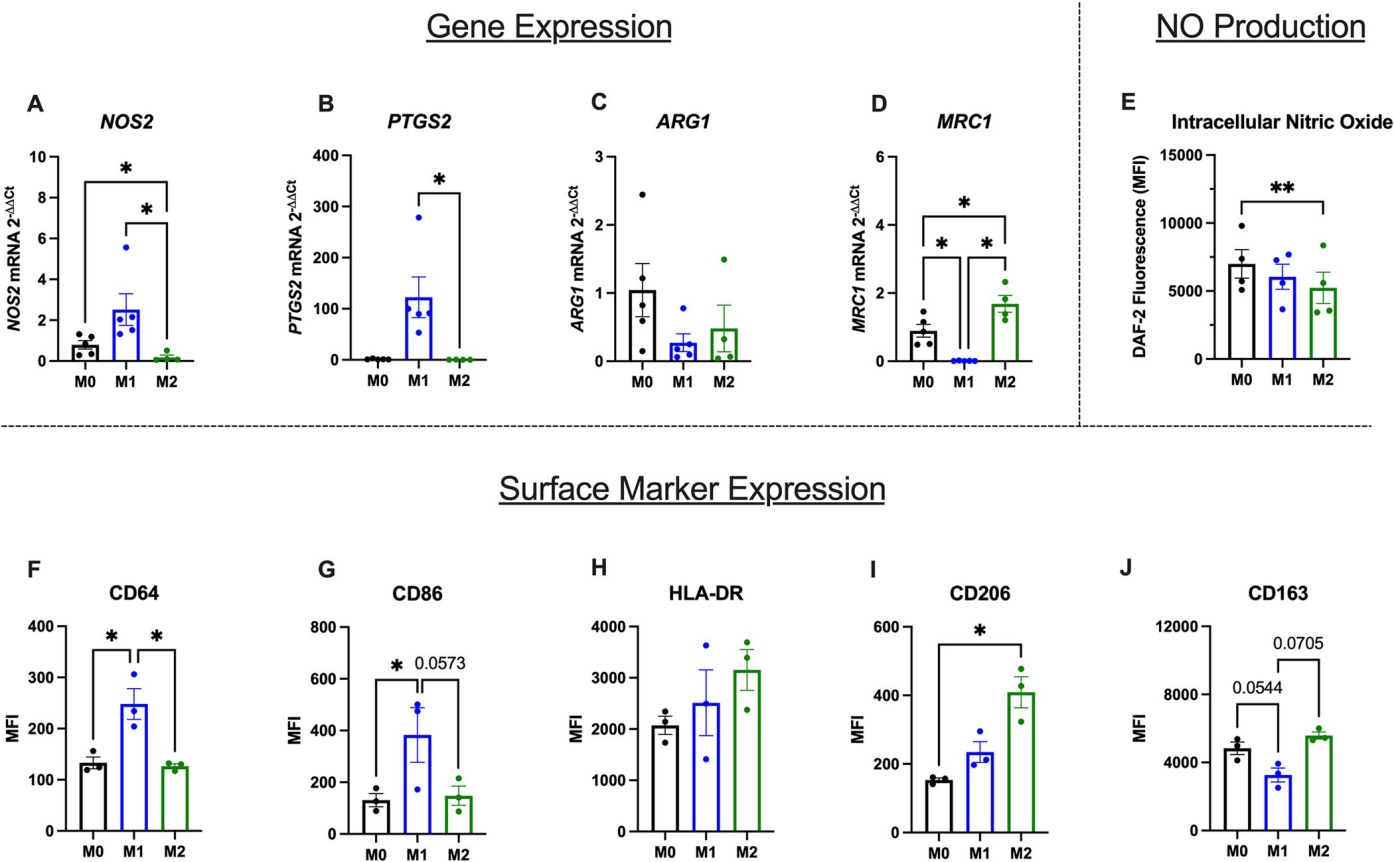
Polarization of hMDMs induces changes in gene expression and cell surface marker expression. hMDM polarization-induced changes in gene expression of (A) NOS2, (B) PTGS2, (C) ARG1, and (D) MRC1 are shown. M1 hMDMs express higher levels of CD64 (E) and CD86 (F) than M0 and M2 hMDMs. There were no significant differences in HLA-DR (G) across polarization states. CD206 expression (H) is significantly increased in M2 hMDMs, and CD163 expression (I) is decreased in M1 hMDMs in comparison to M0- and M2 hMDMs. N = 5 biological replicates (3 males, 2 females for M0 and M1; RNA extraction failed on one M2 sample, resulting in 3 males and 1 female for the M2 group) for gene expression data and n = 3 biological replicates (all males) for cell surface marker data. For both gene and cell surface maker data, samples were pooled from two technical replicates per biological replicate and polarization state. All data are presented as mean ± SEM. For gene expression data, * p < 0.05 by Friedman test with Holm-Sidak multiple comparisons test (which allows for missing values). For cell surface marker data, *p < 0.05 by repeated measures one-way ANOVA with Tukey’s test for multiple comparisons.

**Fig 6:**
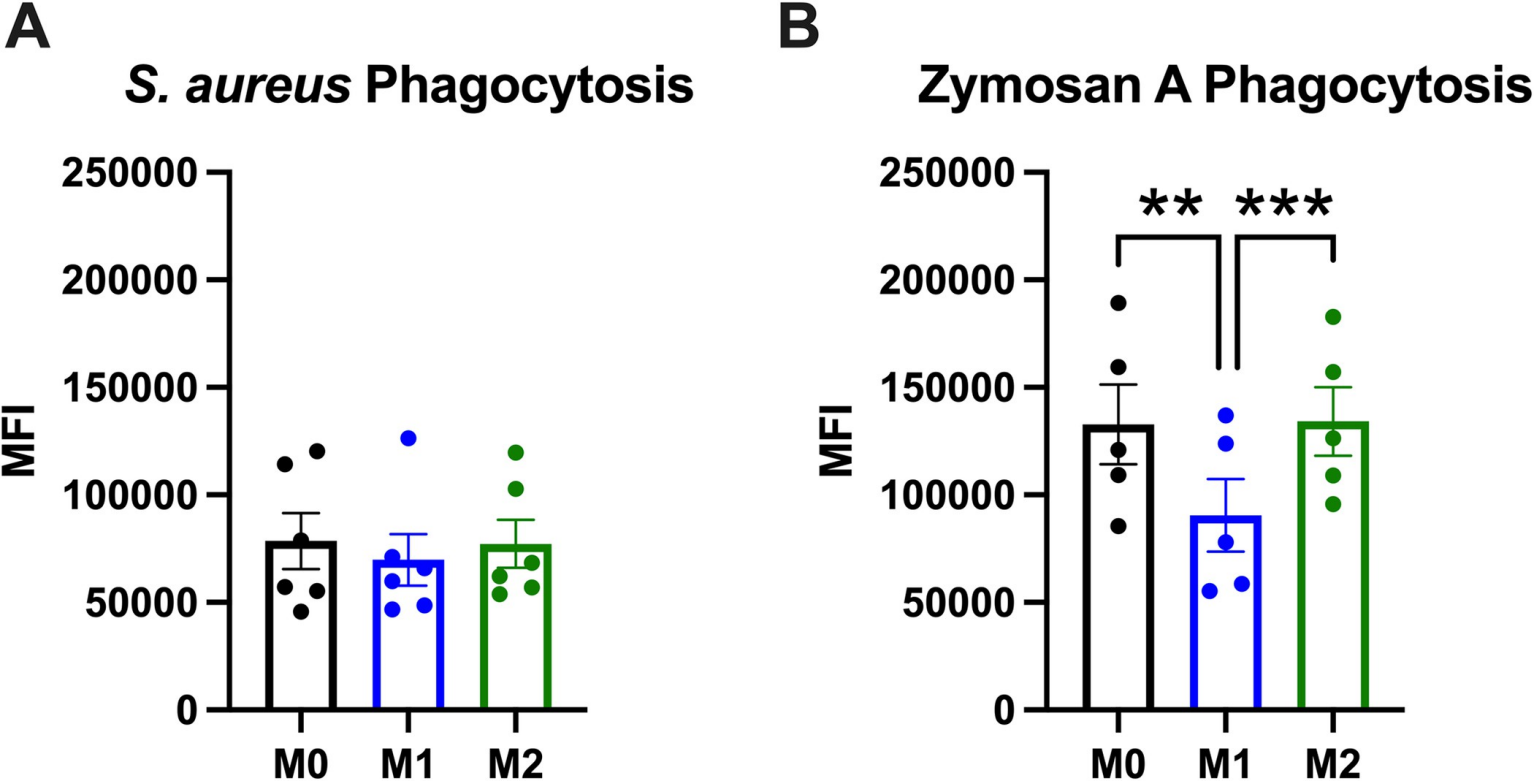
Polarized hMDMs are phagocytic, and M1 hMDMs are significantly less phagocytic of Zymosan A pHrodo Red BioParticles than M0 or M2 hMDMs. hMDMs were assayed for phagocytosis of (A) S. aureus and (B) Zymosan A pHrodo Red BioParticles over two hours. n = 6 biological replicates (3 males, 3 females) for Fig 5A and n = 5 biological replicates (2 males, 3 females) for Fig 5B, with 3 technical replicates per biological replicate and polarization state. Data are presented as mean ± SEM. **p < 0.01, ***p< 0.001 by repeated measures one-way ANOVA with Tukey’s test for multiple comparisons.

To provide a robust panel of endpoints assessing polarization and ensure integration of our novel data with previously demonstrated hMDM phenotypes, we also measured gene expression, cell surface marker expression, and phagocytosis following polarization (Figs 5 and 6). We measured the expression of four genes that have previously been shown to be modulated in response to M1 and M2 polarization (Fig 5) and found that M1 hMDMs had significantly higher expression of *NOS2* in comparison with M0 and M2 hMDMs and significantly higher *PTGS2* expression in comparison with M2 hMDMs (Fig 5A and 5B). We did not detect any significant differences in *ARG1* between polarization conditions (Fig 5C). Expression of *MRC1*, the gene that encodes CD206, was significantly increased in M2 hMDMs and significantly decreased in M1 hMDMs (Fig 5D). This increase in *MRC1* gene expression in M2 hMDMs mirrors the increase in CD206 expression measured with flow cytometry. However, *MRC1* expression in M0 and M1 like hMDMs did not follow the same pattern observed in CD206 surface expression. Overall, these findings agree with previous studies that evaluated gene expression changes in M1 and M2 polarized macrophages *in vitro* [14, 16, 18].

Because we observed a significant increase in *NOS2* expression in M1 hMDMs and decrease in M2 hMDMs in comparison with M0 hMDMs, we next wanted to determine whether nitric oxide production was significantly different across polarization states. We found that M2 hMDMs had significantly lower intracellular nitric oxide in comparison with M0 hMDMs, paralleling gene expression changes (Fig 5E). However, we did not observe a significant increase in intracellular nitric oxide levels in M1 hMDMs, suggesting that the observed gene expression change did not result in a functional change in intracellular nitric oxide.

We then measured the expression of cell surface markers that have been assessed previously in the context of macrophage phenotyping. Using flow cytometry, we found that M1 hMDMs had significantly higher expression of CD64 than M0 and M2 hMDMs and CD86 than M0 hMDMs (Fig 5F and 5G). There was no significant difference in HLA-DR expression between the polarization states (Fig 5H). As expected, M2 hMDMs expressed significantly more CD206 than M0 hMDMs (Fig 5I), and M1 hMDMs expressed less CD163 than M0 and M2 hMDMs, though this difference did not reach statistical significance (Fig 5J). Overall, our results are in agreement with previously published studies assessing cell surface marker expression following M1 and M2 hMDM polarization [14, 18, 40].

Lastly, we wanted to confirm that the polarized hMDMs we generated were phagocytic and determine if there were baseline differences in phagocytosis between polarization states. To determine phagocytic capacity of the polarized hMDMs we used pHrodo Red *S*. *aureus* and zymosan A BioParticles to test bacterial and fungal phagocytosis, respectively. We found that all polarization states had a similar phagocytic capacity for *S*. *aureus* (bacterial) BioParticles (Fig 6A). M1 hMDMs had significantly lower phagocytic capacity for zymosan A (fungal) BioParticles than M0- and M2 hMDMs (Fig 6B). These data agree with previous studies demonstrating increased phagocytosis of zymosan and acidification of the phagolysosome in M2 macrophages [41, 42].

## Discussion

**Fig 7:**
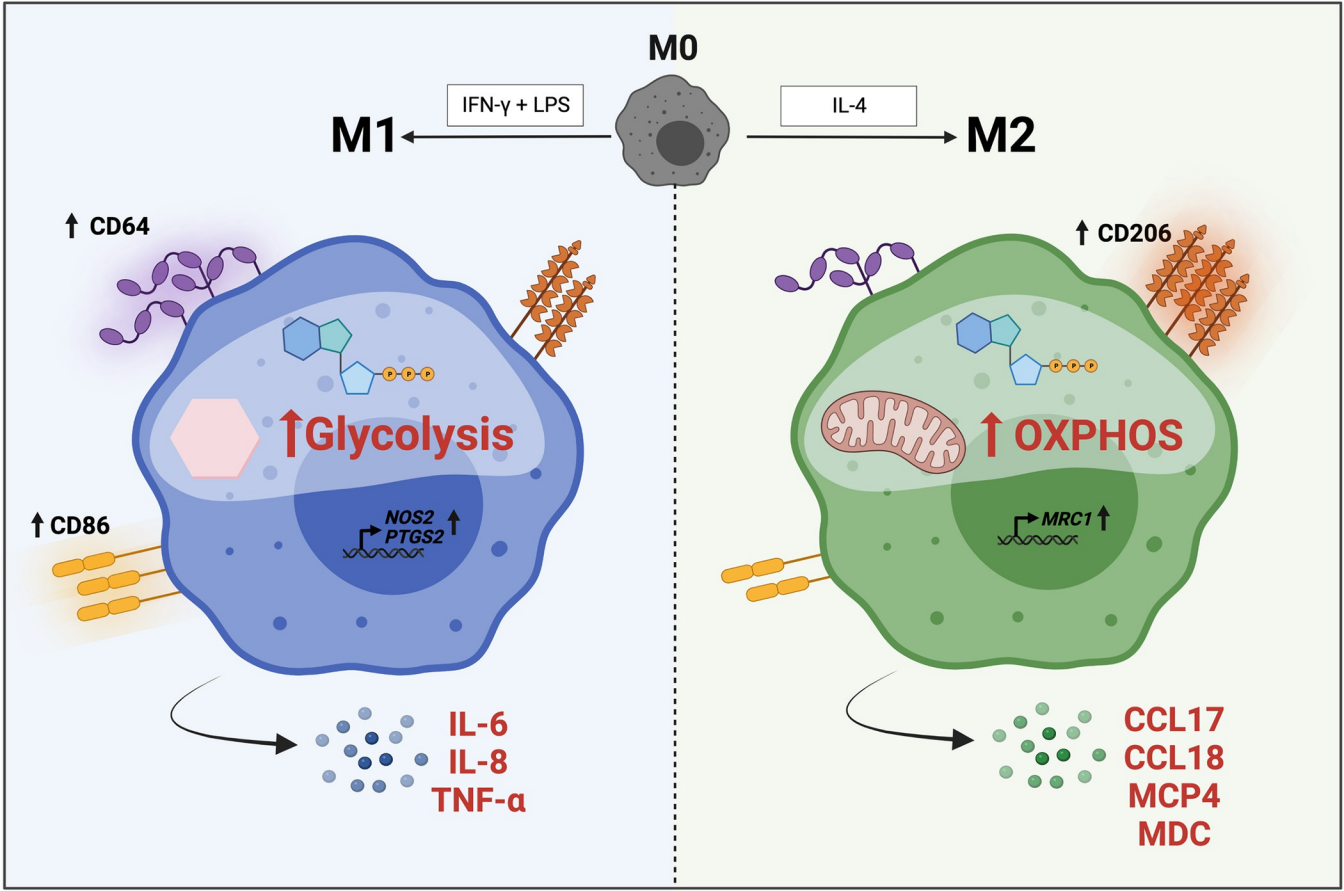
Summary of M1 and M2 hMDM phenotypes. Created with biorender.com.

This study was designed to add cellular bioenergetics and an expanded cytokine profile to the phenotype characterization of hMDMs and their polarization into M1- and M2-like subsets (Fig 7). These new features were integrated with known phenotype changes we observed in cell surface marker and gene expression, reported previously [14, 18, 40]. Our cytokine analysis revealed a unique cluster of soluble mediators (MCP4, MDC, and TARC) secreted by M2-like subsets, while M1-like macrophages secreted significantly more of prototypic pro-inflammatory cytokines (MCP1, eotaxin, eotaxin-3, IL12p70, IL-1α, IL15, TNF-β, IL-6, TNF-α, IL12p40, IL-13, and IL-2). Interestingly, the bioenergetic profiles indicate that M1 hMDMs rely more on glycolysis and exist in an energized, activated state, while the M2 hMDMs depend more on oxidative phosphorylation for their ATP generation. Importantly, the M1 and M2 bioenergetic profiles we observed *in vitro* were similar to bioenergetic profiles we previously observed in healthy humans *in vivo* for sputum (M1) and BAL (M2)-derived macrophages [17].

Because cellular bioenergetics is thought to be a central regulator of macrophage function and downstream engagement of innate and acquired immune cells, and because most previous bioenergetic work has been performed using mouse bone-marrow-derived macrophages (mBMDMs) [18–20, 40], we investigated the important question of how polarization shifts hMDM bioenergetic profiles using Seahorse Extracellular Flux assays. We found that our polarization protocol significantly shifted cellular bioenergetics of hMDMs, with M1 hMDMs significantly more glycolytic than naïve hMDMs (M0) and M2 hMDMs. Specifically, our data showed that spare capacity, coupling efficiency, glycolysis, and proton leak contributed the most to separation of M1 versus M0 and M2 hMDMs (Fig 3B) resulting in M1 hMDMs having less ability to respond to increased energy demand via mitochondrial respiration. Our findings are in agreement with a previous proteomic study of M1 and M2 hMDMs, which demonstrated upregulation of a marker of gluconeogenesis in M1 cells [43], supporting an association between polarization state and bioenergetic shifts. Overall, our bioenergetic findings also agree in part with data presented in studies using mBMDMs in that the M1 hMDMs were more glycolytic than M0 or M2 [18, 20]. However, unlike mBMDMs, our M1 hMDMs were much more responsive to mitochondrial inhibitors [18–20, 44], and our M0 and M2 hMDMs exhibited more significantly reduced glycolytic parameters than previously reported in mBMDMs [18]. These lines of evidence support the previously proposed concept that mechanisms regulating macrophage polarization and bioenergetics are divergent between species and that, while of immense utility, polarized hMDMs display unique features not observed in mBMDMs [40].

Importantly, as surrogates of human macrophages from the respiratory tract, the bioenergetic profiles we observed in our polarized hMDMs were similar to the bioenergetic profiles of sputum and BAL-derived macrophages [17]. M1 hMDMs shared a similar bioenergetic profile to induced sputum macrophages, with significantly lower maximal respiration and higher glycolytic capacity than M2 hMDMs, which had a bioenergetic profile more similar to BAL macrophages [17]. These divergent bioenergetic profiles *in vivo* suggest distinct functions of macrophage subsets depending on their location in the respiratory tract. The presence of M1-like, highly glycolytic macrophages on the surfaces of the large airways confers an advantage to the host airway to quickly respond to the constant presence of inhaled pathogens, while M2-like macrophages remain more bioenergetically quiescent in the pathogen-protected distal regions of the airways mediating homeostasis.

For other endpoints, comparisons between our hMDMs raised in culture and lung macrophages recovered from human volunteers are difficult given that there are few studies that have directly compared these specific endpoints in different subpopulations of human respiratory macrophages. Studies evaluating surface marker expression and gene expression of different human lung macrophage populations have revealed a high degree of complexity in expression patterns and have demonstrated that even within regions of the airways, distinct subpopulations exist [5, 45, 46]. For example, although the mannose receptor CD206, which mediates endocytosis and phagocytosis of mannoglycoprotein-expressing microorganisms and debris, has generally been considered a marker of M2 macrophages, CD206 is expressed on the surface of both alveolar and tissue resident macrophages and is co-expressed with markers associated with M1 polarization, such as HLA-DR [5, 47, 48].

The plasticity of naïve macrophages in different organ systems and their ability to display different phenotypes with vastly different characteristics is an area of intense investigation. While polarization of macrophages during an infection towards a pro-inflammatory M1-like phenotype is a critical component of the innate immune defense response, over exuberant activation can lead to tissue injury. M2-like macrophages have wound-healing and inflammation-resolving capabilities, required to facilitate repair and resolution of inflammation. However, tumors and certain infections can create an environment favoring the wound-healing M2-like phenotype, which allows cancer and infection to progress [49, 50]. Hence, repolarization or “re-educating” macrophages is an area of targeted therapy development. Phagocytosis, chemotaxis/migration, and cytokine release are all ATP-dependent processes that require a rapid adaptation to the increased energy demand, which can be achieved by glycolytic metabolism. In contrast, wound healing depends on a more constant and overall greater energy supply, which is accomplished by oxidative phosphorylation [51]. Emerging data supports the notion that exposure of macrophages to pharmacologic agents that shift mitochondrial metabolism is closely linked to changes in activation states [52]. Hence, a more thoroughly characterized hMDM, M1 and M2 phenotype as described here provides a useful *in vitro* model to examine potential therapeutic or toxic agents that impact macrophage immunometabolism, both as a surrogate for human respiratory tract macrophages and macrophages/monocytes in other organs. This model could also be useful for studying macrophage plasticity associated with toxicants and other stimuli.

In addition to expanding the phenotype characterization of hMDMs with bioenergetic analysis, we included an expanded soluble mediator panel to assess macrophage differentiation and polarization. We found clear differences in mediator secretion in comparison with M0 hMDMs and revealed specific clustering of hMDM phenotypes via principal component analysis performed on mediator data. Our principal component analysis showed mediator clusters that distinguished M2 (MDC, MCP4, TARC/CCL17) from M0 (IL-7, MIP-1⍺, IL-5) from M1 (IL-6, IL-8, TNF-α, IL-10, IL-15) phenotypes. Mediators that distinguished M2 hMDMs generally function as chemoattractants for T lymphocytes and monocytes, while mediators that distinguished M1 hMDMs generally function as immune-cell-activating, proinflammatory signals and/or neutrophil chemoattractants, with the exception of IL-10, commonly considered anti-inflammatory. Understanding clustering of these mediators is a useful tool that could be used as biomarkers and applied to understanding shifts in macrophage populations associated with disease states or toxicant exposure.

One limitation of our study is that we did not investigate the effects of glycolytic inhibitors on macrophage polarization, which would provide additional mechanistic insights given the bioenergetic shifts we observed following polarization. Previous studies suggest that inhibition of glycolysis with 2-deoxyglucose can impair M2 polarization, though these studies were conducted using mBMDMs (reviewed in [53]). One study evaluated the effects of 2-DG on LPS-stimulated hMDM and mBMDM viability and mitochondrial membrane potential [40]; however, additional studies are needed to evaluate whether and how inhibition of glycolysis during and after polarization affects hMDM cellular phenotypes. Given the divergent bioenergetic profiles of our M1 and M2 hMDMs, we hypothesize that inhibiting glycolysis, particularly in M1 cells, would result in altered gene and cell surface marker expression and reflect a phenotype more similar to M2 hMDMs. Additionally, although we observed significant differences in phagocytosis and intracellular nitric oxide production between polarization states, bacterial killing is a complex process, and some bacteria have evolved mechanisms to evade killing [54]. Therefore, additional experiments are needed to further investigate whether there are also significant differences in microbicidal capacity between polarization states by performing experiments with live bacteria.

Overall, our findings add an important and to date missing element, namely bioenergetic profiling, to the characterization of differentiated and polarized hMDMs. Our data suggest that polarized hMDMs provide an acceptable *in vitro* model to study specific macrophage subtypes, which can be extrapolated to human respiratory macrophages specifically based on previous clinical data; however, this polarized macrophage model may also be useful for interrogating macrophage function in other organs. Specifically, it provides an integrated model to study how changes in cellular metabolism are linked to previously observed changes in macrophage function, cell surface markers, and gene expression. However, it is critical to acknowledge that these *in vitro* models do not fully recapitulate the phenotypic diversity and plasticity of human macrophages *in vivo* [55, 56]. For example, larger diversity of macrophage subtypes (M2a, M2b, M2c, M2d) are being uncovered by single cell RNAseq approaches [57, 58] which indicates the need for more diverse *in vitro* models in order to investigate how disease states and inhalational perturbations (e.g. microbes, toxicant exposures) alter human macrophage plasticity and polarization.

## Supporting information

S1 Fig Representative flow cytometric data for one PBMC donor demonstrating enrichment of CD14^+^ monocytes following negative selection. (TIF)

S2 Fig hMDM polarization does not significantly alter mitochondrial membrane potential. Mitochondrial membrane potential was quantified using JC-1 dye. CCCP, which dissipates mitochondrial membrane potential, was used as a positive control (A). There were no significant differences in the ratio of JC-1 red to green fluorescent between polarization states (B). Data in (A) are presented as matched pairs per polarization state and donor (e.g., Donor 1 M1 JC-1 is paired with Donor 1 M1 JC-1 + CCCP). **** p < 0.0001 by paired t-test. Data in (B) are presented as mean ± SEM. Data were analyzed using one-way ANOVA with Tukey’s test for multiple comparisons. N = 4 (2 males, 2 females).
(TIF)

S3 Fig hMDM polarization does not significantly affect cell viability. Cell viability was assayed using CellTox Green. Data are expressed as a percentage of the fluorescence from the lysed cell positive control, representing 100% cytotoxicity. Data are presented as mean ± SEM. * p < 0.05 by Friedman test with Dunn’s multiple comparisons test. N = 4 (2 males, 2 females).
(TIF)

S1 Table Concentrations of secreted mediators commonly assessed following hMDM polarization in pg/mL were measured using ELISA. Concentrations are reported as mean (standard error). n = 6 (3 males, 3 females). ^a^ at least p < 0.05 in comparison with M0; ^b^ at least p < 0.05 in comparison with M1; ^c^ at least p < 0.05 in comparison with M2 by either one-way ANOVA with Tukey’s multiple comparisons test or Friedman test with Dunn’s multiple comparisons test.
(DOCX)

S2 Table Concentrations of mediators secreted by hMDMs in pg/mL were measured using multiplex ELISA. Concentrations are reported as mean (standard error). n = 4 subjects (1 male, 3 females). ^a^ at least p < 0.05 in comparison with M0; ^b^ at least p < 0.05 in comparison with M1; ^c^ at least p < 0.05 in comparison with M2 by either one-way ANOVA with Tukey’s multiple comparisons test or Friedman test with Dunn’s multiple comparisons test.
(DOCX)
