# Supplementary figures and images for: Expanded characterization of in vitro polarized M0, M1, and M2 human monocyte-derived macrophages: Bioenergetic and secreted mediator profiles
Source: PLoS One. 2023 Mar 2;18(3):e0279037. doi: 10.1371/journal.pone.0279037 (PMC9980743; doi:10.1371/journal.pone.0279037)

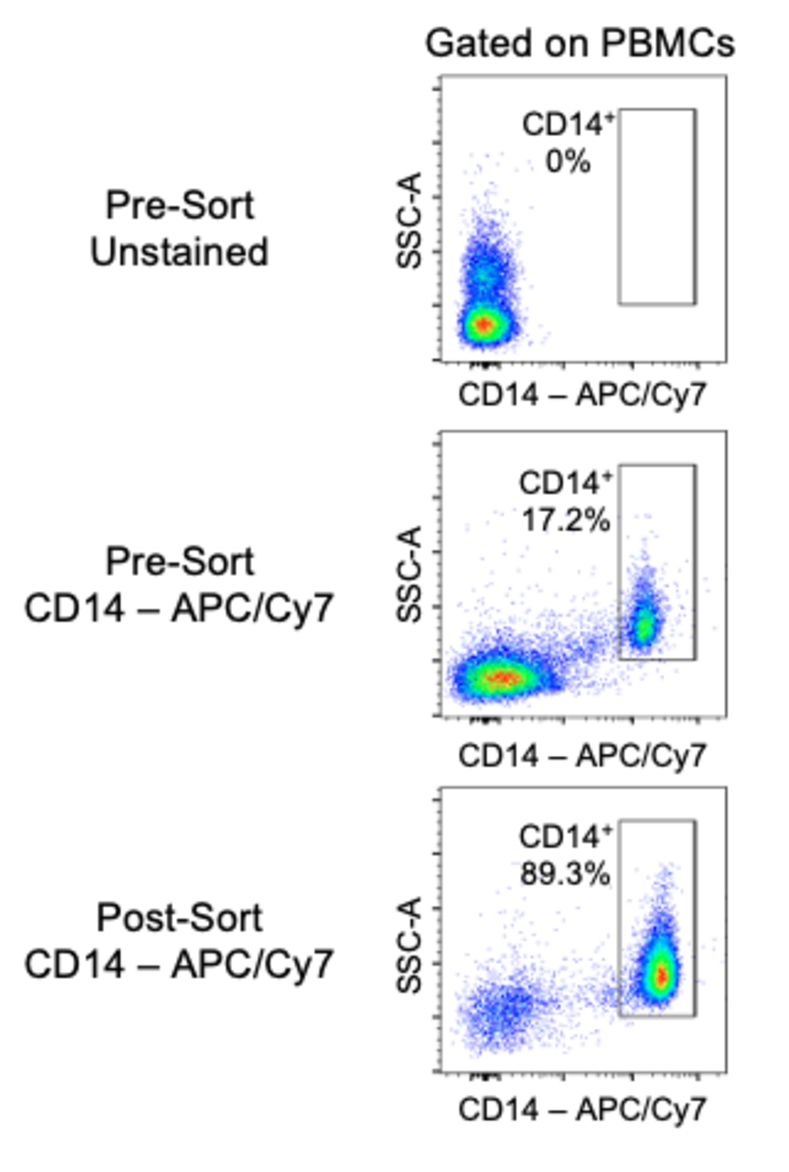

Supplement: S1 Fig — (TIF) [file pone.0279037.s001.tif]

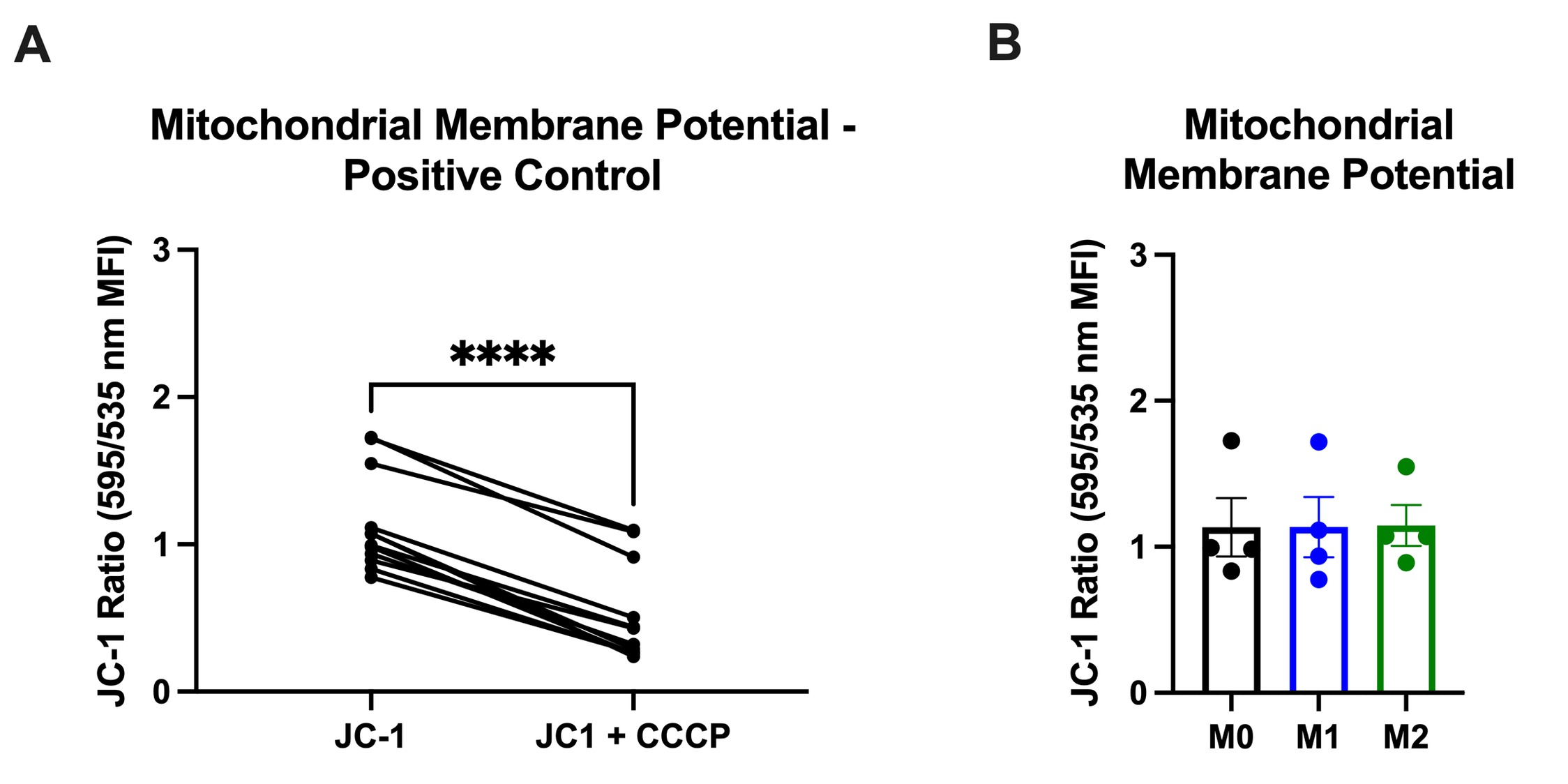

Supplement: S2 Fig — Mitochondrial membrane potential was quantified using JC-1 dye. CCCP, which dissipates mitochondrial membrane potential, was used as a positive control (A). There were no significant differences in the ratio of JC-1 red to green fluorescent between polarization states (B). Data in (A) are presented as matched pairs per polarization state and donor (e.g., Donor 1 M1 JC-1 is paired with Donor 1 M1 JC-1 + CCCP). **** p < 0.0001 by paired t-test. Data in (B) are presented as mean ± SEM. Data were analyzed using one-way ANOVA with Tukey’s test for multiple comparisons. N = 4 (2 males, 2 females). (TIF) [file pone.0279037.s002.tif]

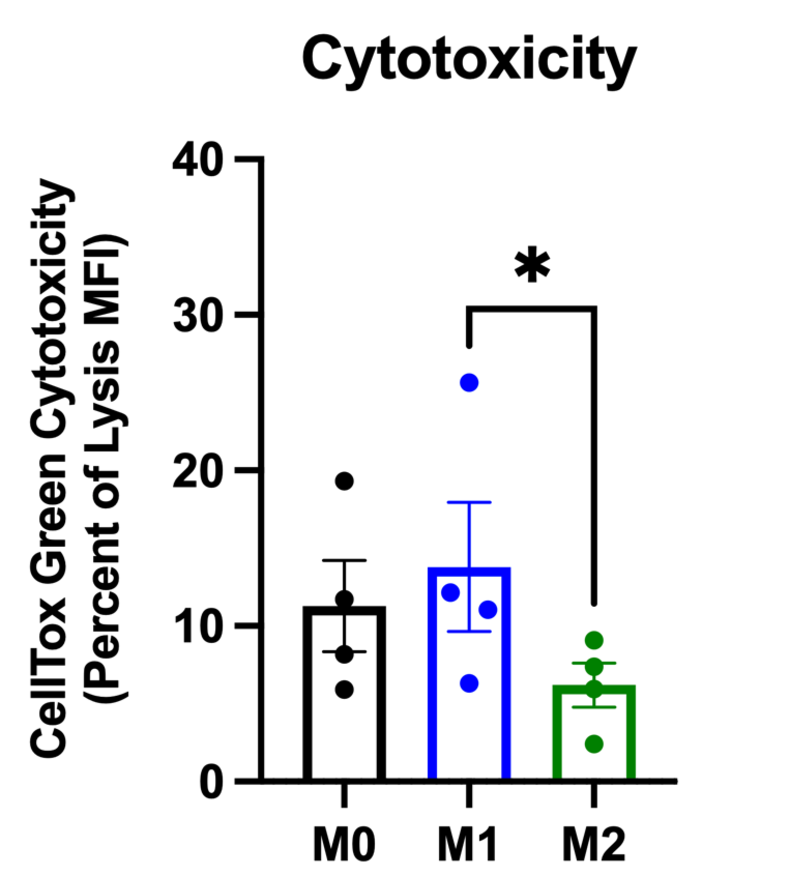

Supplement: S3 Fig — Cell viability was assayed using CellTox Green. Data are expressed as a percentage of the fluorescence from the lysed cell positive control, representing 100% cytotoxicity. Data are presented as mean ± SEM. * p < 0.05 by Friedman test with Dunn’s multiple comparisons test. N = 4 (2 males, 2 females). (TIF) [file pone.0279037.s003.tif]
