# Supplementary material for: Expanded characterization of in vitro polarized M0, M1, and M2 human monocyte-derived macrophages: Bioenergetic and secreted mediator profiles
Source: PLoS One. 2023 Mar 2;18(3):e0279037. doi: 10.1371/journal.pone.0279037 (PMC9980743; doi:10.1371/journal.pone.0279037)
Supplement: S1 Table — Concentrations are reported as mean (standard error). n = 6 (3 males, 3 females). a at least p < 0.05 in comparison with M0; b at least p < 0.05 in comparison with M1; c at least p < 0.05 in comparison with M2 by either one-way ANOVA with Tukey’s multiple comparisons test or Friedman test with Dunn’s multiple comparisons test. (DOCX) [file pone.0279037.s004.docx]

**Table S1. Concentrations of secreted mediators commonly assessed following hMDM polarization in pg/mL were measured using ELISA.**

|  | **M0** | **M1** | **M2** |
| --- | --- | --- | --- |
| **CCL17** | 6.4 (2.8) ^c^ | 6.6 (3.0) ^c^ | 339 (113) ^a,b^ |
| **CCL18** | 215 (47) ^c^ | 1522 (370) | 18801 (4998) ^a^ |
| **IL-6** | 31.9 (9.6) ^b^ | 22167 (4898) ^a,c^ | 13.1 (4.9) ^b^ |
| **IL-8** | 20700 (5300) ^b^ | 164000 (22710) ^a,c^ | 19580 (10200) ^b^ |
| **MMP-9** | 1448000 (100200) ^b^ | 965000 (52400) ^a^ | 1181000 (126100) |
| **TNF-α** | 20.6 (6.7) ^b^ | 28279 (3464) ^a,c^ | 7.8 (0.0) ^b^ |

Concentrations are reported as mean (standard error). n =6 biological replicates (3 males, 3 females) with 2 technical replicates per biological replicate and polarization state. ^a^ at least p < 0.05 in comparison with M0; ^b^ at least p < 0.05 in comparison with M1; ^c^ at least p < 0.05 in comparison with M2 by either one-way ANOVA with Tukey’s multiple comparisons test or Friedman test with Dunn’s multiple comparisons test.
