# Supplementary material for: Expanded characterization of in vitro polarized M0, M1, and M2 human monocyte-derived macrophages: Bioenergetic and secreted mediator profiles
Source: PLoS One. 2023 Mar 2;18(3):e0279037. doi: 10.1371/journal.pone.0279037 (PMC9980743; doi:10.1371/journal.pone.0279037)
Supplement: S2 Table — Concentrations are reported as mean (standard error). n = 4 subjects (1 male, 3 females). a at least p < 0.05 in comparison with M0; b at least p < 0.05 in comparison with M1; c at least p < 0.05 in comparison with M2 by either one-way ANOVA with Tukey’s multiple comparisons test or Friedman test with Dunn’s multiple comparisons test. (DOCX) [file pone.0279037.s005.docx]

**Table S2. Concentrations of mediators secreted by hMDMs in pg/mL were measured using multiplex ELISA.**

|  | **M0** | **M1** | **M2** |
| --- | --- | --- | --- |
| **Eotaxin** | 94.5 (7.92)^b^ | 209 (14.7)^a,c^ | 110 (2.32)^b^ |
| **Eotaxin-3** | 41.5 (3.27)^b^ | 207 (30.1)^a^ | 250 (78.6) |
| **GM-CSF** | 0.486 (0.152) | 20.0 (6.74) | 0.580 (0.229) |
| **IL-1α** | 0.180 (0.180)^b^ | 15.9 (9.19)^a^ | 0.825 (0.379) |
| **IL-1β** | 4.13 (0.274) | 228 (159)^c^ | 2.72 (0.857)^b^ |
| **IL-2** | 5.57 (0.139)^b^ | 16.6 (1.80)^a,c^ | 3.34 (1.19)^b^ |
| **IL-5** | 0.119 (0.0824) | 0.106 (0.0556) | 0.0110 (0.00652) |
| **IL-6** | 12.5 (3.17)^b^ | 2430 (292)^a,c^ | 13.9 (3.43)^b^ |
| **IL-7** | 0.436 (0.0850) | 0.200 (0.0899) | 0.423 (0.0193) |
| **IL-8** | 9770 (1390) | 111000 (30000)^c^ | 7320 (2130)^b^ |
| **IL-10** | 5.77 (1.12) | 1300 (384) | 4.62 (1.15) |
| **IL-12p40** | 0.306 (0.117)^b^ | 577 (184)^a^ | 1.09 (0.534) |
| **IL-12p70** | 2.67 (0.397)^b^ | 15.6 (2.32)^a^ | 4.61 (1.03) |
| **IL-13** | 60.2 (4.38)^b^ | 90.5 (6.29)^a^ | 46.7 (11.3) |
| **IL-15** | 0.517 (0.0853)^b^ | 5.33 (0.494)^a,c^ | 0.359 (0.0484)^b^ |
| **IL-16** | 16.6 (4.31) | 50.7 (17.0) | 32.2 (15.5) |
| **IL-17** | 0 (0) | 2.77 (1.38) | 0.0152 (0.0152) |
| **IP-10** | 315 (153) | 18700 (5360) | 550 (231) |
| **MCP-1** | 4000 (70.0) | 4100 (51.5) | 4060 (76.1) |
| **MCP-4** | 54.4 (4.21)^b,c^ | 106 (9.04)^a,c^ | 3110 (334)^a,b^ |
| **MDC** | 1330 (579) | 1070 (214) | 14900 (8490) |
| **MIP-1α** | 80.0 (6.44) | 1830 (1830) | 305 (100) |
| **MIP-1β** | 384 (207) | 11700 (3200) | 2010 (1070) |
| **TARC** | 22.0 (9.23)^c^ | 53.7 (5.69) | 1070 (505)^a^ |
| **TNF.α** | 14.0 (2.11)^b^ | 3260 (33.1)^a,c^ | 17.4 (6.49)^b^ |
| **TNF.β** | 0.0300 (0.0300)^b^ | 4.84 (2.92)^a^ | 0.0912 (0.0372) |
| **VEGF** | 43.1 (17.7) | 514 (129)^c^ | 5.40 (2.17)^b^ |

Concentrations are reported as mean (standard error). n = 4 biological replicates (1 male, 3 females) with one technical replicate per biological replicate and polarization state. ^a^ at least p < 0.05 in comparison with M0; ^b^ at least p < 0.05 in comparison with M1; ^c^ at least p < 0.05 in comparison with M2 by either one-way ANOVA with Tukey’s multiple comparisons test or Friedman test with Dunn’s multiple comparisons test.
